# Supplementary material for: Systematic design of pulse dosing to eradicate persister bacteria
Source: PLoS Comput Biol. 2023 Jan 17;19(1):e1010243. doi: 10.1371/journal.pcbi.1010243 (PMC9882918; doi:10.1371/journal.pcbi.1010243)
Supplement: S1 Text — Appendix A. Derivation of Eq (7); Appendix B. Optimal rate of decline for bacterial population peaks characterized by Eq (9); Appendix C. Estimation of Kn,off, Kn,on from data in Fig 5; Appendix D. Analytical solution of Eqs (1) and (2). (DOCX) [file pcbi.1010243.s001.docx]

Systematic Design of Pulse Dosing to Eradicate Persister Bacteria

Systematic Design of Pulse Dosing to Eradicate Persister Bacteria

Garima Singh, Mehmet A. Orman, Jacinta C. Conrad, Michael Nikolaou^*^

Chemical and Biomolecular Engineering Department,

University of Houston, Houston, TX, United States

*****Corresponding Author:
nikolaou@uh.edu

**Supporting information**

#### APPENDIX A. Derivation of eqn. (7)

Consider pulse dosing with antibiotic switched on ($C\neq0$) and off ($C=0$) at times $t_{0}, t_{1}, t_{2}, t_{3},\ldots$ and with respective pulse durations $t_{\text{on}},t_{\text{off}}.$ Then, eqn. (3) implies

$\mathbf{x}\left( t_{1} \right)=\exp\left( \mathbf{A}_{\text{on}}t_{\text{on}} \right)\mathbf{x}\left( t_{0} \right)$

$\mathbf{x}\left( t_{2} \right)=\exp\left( \mathbf{A}_{\text{off}}t_{\text{off}} \right)\mathbf{x}\left( t_{1} \right)=\exp\left( \mathbf{A}_{\text{off}}t_{\text{off}} \right)\exp\left( \mathbf{A}_{\text{on}}t_{\text{on}} \right)\mathbf{x}\left( t_{0} \right)$

$\mathbf{x}\left( t_{3} \right)=\ldots=\exp\left( \mathbf{A}_{\text{on}}t_{\text{on}} \right)\exp\left( \mathbf{A}_{\text{off}}t_{\text{off}} \right)\exp\left( \mathbf{A}_{\text{on}}t_{\text{on}} \right)\mathbf{x}\left( t_{0} \right)$

$\vdots$

$\mathbf{x}\left( t_{2\mathcal{l}} \right)$

$=\underset{\mathcal{l}}{\underbrace{\left( \overset{\mathbf{M}}{\overbrace{\exp\left( \mathbf{A}_{\text{off}}t_{\text{off}} \right)\exp\left( \mathbf{A}_{\text{on}}t_{\text{on}} \right)}} \right)\ldots\left( \overset{\mathbf{M}}{\overbrace{\exp\left( \mathbf{A}_{\text{off}}t_{\text{off}} \right)\exp\left( \mathbf{A}_{\text{on}}t_{\text{on}} \right)}} \right)}}\mathbf{x}\left( t_{0} \right)$

$=\mathbf{M}^{\mathcal{l}}\mathbf{x}\left( t_{0} \right), \mathcal{l}=0, 1, 2,\ldots$

and

$\mathbf{x}\left( t_{2\mathcal{l}+1} \right)$

$=\exp\left( \mathbf{A}_{\text{on}}t_{\text{on}} \right)\underset{\mathcal{l}}{\underbrace{\left( \overset{\mathbf{M}}{\overbrace{\exp\left( \mathbf{A}_{\text{off}}t_{\text{off}} \right)\exp\left( \mathbf{A}_{\text{on}}t_{\text{on}} \right)}} \right)\ldots\left( \overset{\mathbf{M}}{\overbrace{\exp\left( \mathbf{A}_{\text{off}}t_{\text{off}} \right)\exp\left( \mathbf{A}_{\text{on}}t_{\text{on}} \right)}} \right)}}\mathbf{x}\left( t_{0} \right)$

$=\exp\left( \mathbf{A}_{\text{on}}t_{\text{on}} \right)\mathbf{M}^{\mathcal{l}}\left( t_{0} \right), \mathcal{l}=0, 1, 2,\ldots$

Therefore

$c\left( t_{2\mathcal{l}} \right)=\mathbf{c}^{\boldsymbol{\top}}\mathbf{M}^{\mathcal{l}}\mathbf{x}\left( t_{0} \right)$ (SI-1)

$c\left( t_{2\mathcal{l}+1} \right)=\mathbf{c}^{\boldsymbol{\top}}\exp\left( \mathbf{A}_{\text{on}}t_{\text{on}} \right)\mathbf{M}^{\mathcal{l}}\mathbf{x}\left( t_{0} \right)$ (SI-2)

where $\mathbf{c}^{\boldsymbol{\top}}\boldsymbol{=}\left[ \begin{matrix} 1 & 1 \end{matrix} \right]$.

The above two equations suggest that peaks and dips of $c(t)$ at times $t_{2\mathcal{l}}$ and $t_{2\mathcal{l}+1}$, respectively, increase or decrease at the same rate, governed by the eigenvalues $\lambda_{1}, \lambda_{2}$ of the matrix

$\mathbf{M≝}\exp\left( \mathbf{A}_{\text{off}}t_{\text{off}} \right)\exp\left( \mathbf{A}_{\text{on}}t_{\text{on}} \right)$ (SI-3)

as

$c\left( t_{2\mathcal{l}} \right)=\mathbf{c}^{\boldsymbol{\top}}\mathbf{M}^{\mathcal{l}}\mathbf{x}\left( t_{0} \right)=\mathbf{c}^{\boldsymbol{\top}}\mathbf{P}\boldsymbol{\Lambda}^{\mathcal{l}}\mathbf{P}^{-1}\mathbf{x}\left( t_{0} \right)$ (SI-4)

and similarly for $c\left( t_{2\mathcal{l+}1} \right)$.

Now, because $a\approx0$, the eigenvalues, $\rho_{1}, \rho_{2}$ of

$\mathbf{A≝}\left[ \begin{matrix} K_{n} & b \\ a & K_{p} \end{matrix} \right]$ (SI-5)

in eqn. (3) are approximately

$\boxed{\rho_{1}\approx K_{n}, \rho_{2}\approx K_{p}}$ (SI-6)

When the antibiotic is on, then $b\approx0$, and

$K_{n,\text{on}}<K_{p,\text{on}}<0$ (SI-7)

with $K_{p,\text{on}}$ closer to 0 than $K_{n,\text{on}}$, because $K_{p,\text{on}}$ refers to persisters, in contrast to $K_{n,\text{on}},$ which refers to normal cells that get killed much faster (e.g. see Table 1). In addition, the corresponding eigenvectors of $\mathbf{A}_{\text{on}}$ are $\mathbf{w}_{\text{1,on}}\boldsymbol{=}\left[ \begin{matrix} 1 & 0 \end{matrix} \right]^{\boldsymbol{\top}}\boldsymbol{,}\mathbf{w}_{\text{2,on}}\boldsymbol{=}\left[ \begin{matrix} 0 & 1 \end{matrix} \right]^{\boldsymbol{\top}}\boldsymbol{.}$ Therefore,

$\exp\left( \mathbf{A}_{\text{on}}t_{\text{on}} \right)=\left[ \begin{matrix} \exp\left( K_{n,\text{on}}t_{\text{on}} \right) & 0 \\ 0 & \exp\left( K_{p,\text{on}}t_{\text{on}} \right) \end{matrix} \right]$ (SI-8)

Similarly, when the antibiotic is off, it follows that

$K_{p,\text{off}}<0<K_{n,\text{off}}$ (SI-9)

because the normal cell subpopulation grows, whereas persister cells decline due to their returning to the state of normal cells (e.g. see Table 1). In addition, the corresponding eigenvectors of $\mathbf{A}_{\text{off}}$ are $\mathbf{w}_{\text{1,off}}\boldsymbol{=}\left[ \begin{matrix} 1 & 0 \end{matrix} \right]^{\boldsymbol{\top}}\boldsymbol{,}\mathbf{w}_{\text{2,off}}\boldsymbol{=}\left[ \begin{matrix} 1 & \xi\end{matrix} \right]^{\boldsymbol{\top}}$ where $\xi=(K_{p}-K_{n})/b$. Therefore

$\exp\left( \mathbf{A}_{\text{off}}t_{\text{off}} \right)=\exp\left( K_{n,\text{off}}t_{\text{off}} \right)\mathbf{w}_{\text{1,off}}\mathbf{z}_{\text{1,off}}^{\boldsymbol{\top}}\boldsymbol{+}\exp\left( K_{p,\text{off}}t_{\text{off}} \right)\mathbf{w}_{\text{2,off}}\mathbf{z}_{\text{2,off}}^{\boldsymbol{\top}}$ (SI-10)

where $\mathbf{z}_{\text{1,off}}^{\boldsymbol{\top}}\boldsymbol{=}\left[ \begin{matrix} 1 & -1/\xi\end{matrix} \right]\boldsymbol{,}\mathbf{z}_{\text{2,off}}^{\boldsymbol{\top}}\boldsymbol{=}\left[ \begin{matrix} 1 & 1/\xi\end{matrix} \right]$ are the rows of the inverse modal matrix.

Combination of the last two equations (SI-10) and (SI-8) with the above definition of $\mathbf{M}$ in eqn. (SI-3) yields

$\mathbf{M}=\left[ \begin{matrix} \exp\left( K_{n,\text{off}}t_{\text{off}}+K_{n,\text{on}}t_{\text{on}} \right) & X \\ 0 & \exp\left( K_{p,\text{off}}t_{\text{off}}+K_{p,\text{on}}t_{\text{on}} \right) \end{matrix} \right]$ (SI-11)

where the actual form of $X$ in the above eqn. (SI-11) does not affect the eigenvalues of $\mathbf{M}$**,** which are

$\boxed{\lambda_{1}=\exp\left( K_{n,\text{off}}t_{\text{off}}+K_{n,\text{on}}t_{\text{on}} \right), \lambda_{2}=\exp\left( K_{p,\text{off}}t_{\text{off}}+K_{p,\text{on}}t_{\text{on}} \right)}$ (SI-12)

For $\mathbf{M}^{\mathcal{l}}$ to decline as $\mathcal{l}$ increases, it is necessary and sufficient that both $0<\lambda_{1}<1$ and $0<\lambda_{2}<1$, which is equivalent to

$K_{n,\text{off}}t_{\text{off}}+K_{n,\text{on}}t_{\text{on}}<0\Leftrightarrow\boxed{\frac{t_{\text{off}}}{t_{\text{on}}}<-\frac{K_{n,\text{on}}}{K_{n,\text{off}}}}$ (SI-13)

and

$K_{p,\text{off}}t_{\text{off}}+K_{p,\text{on}}t_{\text{on}}<0\Leftrightarrow\frac{t_{\text{off}}}{t_{\text{on}}}>-\frac{K_{p,\text{on}}}{K_{p,\text{off}}}$ (SI-14)

in view of the inequalities in eqns. (SI-7) and (SI-9).

The inequality in eqn. (SI-14) is trivially satisfied, given eqns. (SI-7) and (SI-9). Therefore, it is the inequality in eqn. (SI-13) that characterizes the feasible region for $t_{\text{off}}/t_{\text{on}}$.

#### APPENDIX B. Optimal rate of decline for bacterial population peaks characterized by eqn. (9)

Eqn. implies that successive peaks of $c\left( t \right)$ at times $t_{2\mathcal{l}}$ (Fig 1) are characterized as

$c\left( t_{2\mathcal{l}} \right)=p_{1}\lambda_{1}^{\mathcal{l}}+p_{2}\lambda_{2}^{\mathcal{l}}, \mathcal{l}=0, 1, 2, \ldots$ (SI-15)

where $\lambda_{1}, \lambda_{2}$ are the eigenvalues of $\mathbf{M}$ and $p_{1}, p_{2}$ are coefficients depending on the model parameters and initial conditions. Each of the two terms $\lambda_{1}^{\mathcal{l}}, \lambda_{2}^{\mathcal{l}}$ in eqn. (SI-15) corresponds to a decline ratio from time point $t_{2\mathcal{l}}$ to $t_{2\left( \mathcal{l}+1 \right)}$. For values of $t_{\text{off}}/t_{\text{on}}$ satisfying eqn. (SI-13), which guarantees $0<\lambda_{1}<1, 0<\lambda_{2}<1$ hence decline of successive peaks, the larger of $\lambda_{1}, \lambda_{2}$ captures the slower of the two modes of decline ratio from peak to peak. The two planes characterized by the inequalities in eqns. (SI-13) and (SI-14) written as equalities (cf. Fig 2) intersect at the straight line $\lambda_{1}=\lambda_{2}$ which yields

$\boxed{\frac{t_{\text{off}}}{t_{\text{on}}}=\frac{K_{p,\text{on}}-K_{n,\text{on}}}{K_{n,\text{off}}-K_{p,\text{off}}}>0}$ (SI-16)

as exemplified in for the parameter values in Table 1.


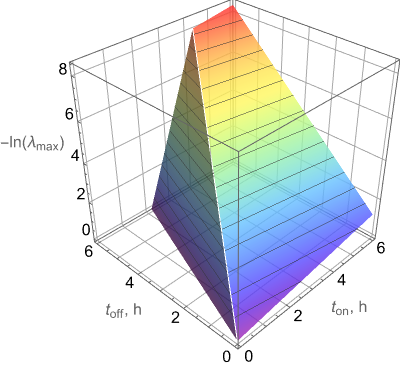


SI Fig 1. Larger (slower) of the two eigenvalues $\lambda_{1}, \lambda_{2}$ of the matrix $\mathbf{M}$ as a function of $t_{\text{on}}, t_{\text{off}}$ for the values of $K_{n,\text{on}}, K_{p,\text{on}}, K_{n,\text{off}}, K_{p,\text{off}}$ in Table 1. The crease line where the two planes intersect corresponds to eqn. and characterizes the highest peak-to-peak decline ratio at discrete time points $t_{2\mathcal{l}}≝\mathcal{l}\left( t_{\text{on}},+t_{\text{off}} \right)$.

The peak-to-peak decline ratio for each mode at discrete time points $t_{2\mathcal{l}}≝\mathcal{l}\left( t_{\text{on}},+t_{\text{off}} \right)$ can be easily converted to actual time rate:

$\lambda^{\mathcal{l}}=\exp\left( \mathcal{l}\ln\left( \lambda\right) \right)=\exp\left( \frac{t_{2\mathcal{l}}}{t_{\text{on}}+t_{\text{off}}}\ln\left( \lambda\right) \right)$ (SI-17)

which, combined with eqn. immediately implies actual-time decline rates

$k_{1}=\frac{\ln\left( \lambda_{1} \right)}{t_{\text{on}}+t_{\text{off}}}=\frac{K_{n,\text{off}}t_{\text{off}}+K_{n,\text{on}}t_{\text{on}}}{t_{\text{on}}+t_{\text{off}}}, k_{2}=\frac{\ln\left( \lambda_{2} \right)}{t_{\text{on}}+t_{\text{off}}}=\frac{K_{p,\text{off}}t_{\text{off}}+K_{p,\text{on}}t_{\text{on}}}{t_{\text{on}}+t_{\text{off}}}$ (SI-18)

or corresponding time constant

$\tau_{1}=\frac{1}{k_{1}}, \tau_{2}=\frac{1}{k_{2}}$ (SI-19)

as indicated in Fig 11.

Note that the values of $k_{1},k_{2}$ or $\tau_{1}, \tau_{2}$ depend on the ratio $\frac{t_{\text{off}}}{t_{\text{on}}}$ rather than on individual values of $t_{\text{on}}, t_{\text{off}}$.

To capture with a single term both modes of peak-to-peak decline corresponding to $\lambda_{1}, \lambda_{2}$ in eqn. (SI-15), one can use the geometric average of $k_{1}, k_{2}$ from eqn. (SI-18), i.e.

$k≝\sqrt{k_{1}k_{2}}=\frac{\sqrt{\ln\left( \lambda_{1} \right)\ln\left( \lambda_{2} \right)}}{t_{\text{on}}+t_{\text{off}}}=\frac{\sqrt{\left( K_{n,\text{off}}t_{\text{off}}+K_{n,\text{on}}t_{\text{on}} \right)\left( K_{p,\text{off}}t_{\text{off}}+K_{p,\text{on}}t_{\text{on}} \right)}}{t_{\text{on}}+t_{\text{off}}}$ (SI-20)

or, equivalently the geometric average of $\tau_{1}, \tau_{2}$ from eqn. (SI-19), both of which yield eqn. (9).

To characterize the maximum of the overall decline rate in eqn. (SI-20), first observe that

$k=\frac{\sqrt{\left( K_{n,\text{off}}x+K_{n,\text{on}} \right)\left( K_{p,\text{off}}x+K_{p,\text{on}} \right)}}{1+x}$ (SI-21)

where $x≝\frac{t_{\text{off}}}{t_{\text{on}}}$. Then the setting $dk/dx=0$ immediately yields

$\left( \frac{t_{\text{off}}}{t_{\text{on}}} \right)_{\text{opt}}=\frac{2K_{n,\text{on}}K_{p,\text{on}}-K_{n,\text{on}}K_{p,\text{off}}-K_{n,\text{off}}K_{p,\text{on}}}{2K_{n,\text{off}}K_{p,\text{off}}-K_{n,\text{on}}K_{p,\text{off}}-K_{n,\text{off}}K_{p,\text{on}}}$ (SI-22)

which is eqn. (11).

#### APPENDIX C. Estimation of $K_{n,\text{off}}, K_{n,\text{on}}$ from data in Fig 5

Assuming that persister cells are initially a tiny minority in the time-growth experiment (antibiotic off), the standard cell-balance equation is

$\frac{dc}{dt}=K_{n,\text{off}}c\left( t \right)\left( 1-\frac{c\left( t \right)}{c_{\text{max}}} \right)\Leftrightarrow c\left( t \right)=c_{0}\exp\left( K_{n,\text{off}}t \right)\frac{1}{1+\frac{c_{0}}{c_{\text{max}}}\left( \exp\left( K_{n,\text{off}}t \right)-1 \right)}$ (SI-23)

Application to the data shown in Fig 5(a) yields

$$\begin{matrix} & \mathrm{Estimate} & Standard Error & & \\ K_{n,\text{off}} & 1.35 & 0.09 & & \\ c_{0} & 2.4\times{10}^{7} & 0.3\times{10}^{7} & & \\ c_{\text{max}} & 1.4\times{10}^{9} & 0.1\times{10}^{9} & & \end{matrix}$$

In the time-kill experiment, eqn. (SI-23) written for a declining population before persister cells take over becomes

$\frac{dc}{dt}=K_{n,\text{on}}c\left( t \right)\Leftrightarrow c\left( t \right)=c_{0}\exp\left( K_{n,\text{on}}t \right)$ (SI-24)

Application to the data shown in Fig 5(b) yields

$$\begin{matrix} & \mathrm{Estimate} & Standard Error & & \\ K_{n,\text{on}} & -3.3 & 1.0 & & \\ \log_{10} c_{0} & 7.5 & 0.8 & & \\ & & & & \end{matrix}$$

#### APPENDIX D. Analytical solution of eqns. (1) and (2)

Given the eigenvalues

$$\rho_{1,2}=\frac{K_{n}+K_{p}}{2}\pm\frac{\sqrt{4ab+\left( K_{n}-K_{p} \right)^{2}}}{2}$$

of the matrix $\mathbf{A}$, yields

$$c\left( t \right)=ⅇ^{\rho_{1}t}\left( \underset{q_{1}}{\underbrace{\frac{n_{0}+p_{0}}{2}+\frac{\left( K_{n}-K_{p} \right)\left( n_{0}-p_{0} \right)/2+an_{0}+bp_{0}}{\sqrt{4ab+\left( K_{n}-K_{p} \right)^{2}}}}} \right)$$

$$+ⅇ^{\rho_{2}t}\left( \underset{q_{2}}{\underbrace{\frac{n_{0}+p_{0}}{2}-\frac{\left( K_{n}-K_{p} \right)\left( n_{0}-p_{0} \right)/2+an_{0}+bp_{0}}{\sqrt{4ab+\left( K_{n}-K_{p} \right)^{2}}}}} \right)$$
