# Supplementary material for: Systematic design of pulse dosing to eradicate persister bacteria
Source: PLoS Comput Biol. 2023 Jan 17;19(1):e1010243. doi: 10.1371/journal.pcbi.1010243 (PMC9882918; doi:10.1371/journal.pcbi.1010243)
Supplement: Attachment — Submitted filename: Response_to_Reviewer_Comments.docx [file pcbi.1010243.s002.docx]

Reviewer's Responses to Questions

**Comments to the Authors:
Please note here if the review is uploaded as an attachment.**

# Reviewer #1:

This is an interesting study providing a computational model for eradicating dormant persister cells by pulse-dosing an antibiotic, and testing theoretical predictions. This reviewer only analyzed the experimental part of the study. The overall agreement between model and experiment is impressive. Specific comments follow.

1. “In fact, the mechanisms governing persister formation, survival, and resuscitation are diverse and not sufficiently understood (Balaban et al., 2013).”
- That was indeed true a decade ago. Since then, understanding of mechanisms governing persister formation improved significantly. See, for example, (Lewis, K., ed., (2019) Persister Cells and Infectious Disease, p. 295. Springer Nature Switzerland AG.)

- Response: We agree with the Reviewer that there has been a lot of progress in the last decade and perhaps the statement “not sufficiently understood” may undermine the value of this progress. In fact, the key message of this sentence lies in the diversity of these mechanisms and the difficulty to easily account for them in a therapeutic exercise, even if a lot of the (diverse) fundamentals are known.
- Action taken: “diverse and not sufficiently understood” has been replaced by “diverse and multifaceted”.
  The reference by Lewis (2019) has been added.

2. “Such development can be broadly classified into two categories: (a) developing new anti-persister drugs, and (b) manipulating the dosing regimen of approved antibiotics.”
- The most common approach is combining approved antibiotics.

- Response: The Reviewer is entirely correct. Development of antibiotics is indeed a lengthy and resource-intensive process. Which is also a main reason we have chosen to look at option (b)
- Action taken: Text added for emphasis of choice (b): “Such development can be broadly classified into two categories: (a) developing new anti-persister drugs, and the most common approach, (b) manipulating the dosing regimen of approved antibiotics...”

3. Fig. 5, treatment with constant administration of ampicillin over 3 hours results in an approximately 3log decrease in viable cells. Fig. 6, an experiment apparently identically designed, constant administration of ampicillin over 3 hours results in an approximately 5log decrease in viable cell. This discrepancy needs to be addressed.

- Response: Both figures show a 5 log decrease, but perhaps they caused a misreading.
- Action taken: Text was added for clarification: “Note that both Figure 5 and Figure 6 indicate a 5 log decrease of viable cells over the first 3 hours.”

# Reviewer #2:

Singh et al proposed a novel theoretical framework to predict the efficacy of pulsed antibiotic dosing in eradicating persister bacteria. I believe that the study is well conceived and it is backed up by a few experimental data. In my opinion the authors will need to address the issues reported below before this manuscript can be published in PLoS Computational Biology.

Abstract
I suggest rewrite/clarifying the following statement: “A key outcome of the theoretical analysis, on which the proposed methodology is based, is that dosing effectiveness depends mainly on the ratio of corresponding pulse durations.” It is not clear what the authors mean with ratio of pulse durations

- Action taken: Text modified as “A key outcome of the theoretical analysis, on which the proposed methodology is based, is that bactericidal effectiveness of periodic pulse dosing depends mainly on the ratio of durations of the corresponding on and off parts of the pulse.”

Introduction
Lines 28-29. The field has moved forward from the notion that persisters must be in a slow growth state. A number of studies (including by one of the authors of the paper) suggest that persisters can also be in a growing state, e.g.:
<https://journals.asm.org/doi/10.1128/AAC.00243-13>
<https://doi.org/10.7554/eLife.74062>
<https://www.embopress.org/doi/full/10.15252/msb.20145949>
The authors should acknowledge these recent findings and amend this statement accordingly.

- Response: Slow growth referring to population. Nevertheless, streamlined.
- Action taken: Introductory sentence modified as “Persister cells are a small fraction of a bacterial population in a physiological state that enables them to survive otherwise lethal doses of antibiotics.”

Lines 40-42: the authors correctly state that “our knowledge of persisters accelerated in the last two decades” but report nothing about most recent findings, e.g.:
<https://www.science.org/doi/full/10.1126/science.aaf4762>
<https://www.sciencedirect.com/science/article/pii/S2211124722001516>
<https://journals.asm.org/doi/10.1128/mBio.00909-21>
<https://www.nature.com/articles/s41467-022-28141-x>
<https://journals.asm.org/doi/full/10.1128/mBio.00703-21>
<https://pubs.acs.org/doi/10.1021/acsinfecdis.1c00154>

- Response: We agree with the Reviewer that these references shed light to the diverse and multifaceted phenomena in persisters. The only exception is the first one, which does address phenotypical changes but in the context of resistance via efflux pumps.
- Action taken: Citations to five of the above references added to the revised manuscript (references 14, 17-20) along with text summarizing their overall contribution as “Persisters survive via a plethora of putative molecular mechanisms [14], and recent studies have started shedding light on how diverse and multifaceted phenomena can link effects of initial states and environmental factors to phenotypic changes during persister formation, survival, and return to normal state [14–20]. In view of that complexity, developing anti-persister therapeutics remains a challenge.”

The authors could also add another phenotype, i.e. viable but non culturable cells, i.e.
doi:10.1128/AAC.00243-13
<https://doi.org/10.1186/s12915-017-0465-4>
10.1016/j.tim.2014.09.004
The framework they have developed could also help in the eradication of this surviving phenotype

- Response: Indeed, viable but not culturable (VBNC) cells are another important phenotype that is hard to study and poses therapeutic challenges when infectious. We believe that the methodology we presented might be successful at eradicating VBNC cells, provided these cells would return to the natural growth state (hence to susceptibility to the antibiotic) upon provision of appropriate stimuli.
- Action taken: The following bullet point has been added to Conclusions and Future Work:
  “Extension to viable but not culturable cells: In addition to persisters, a well known bacterial phenotype that can survive exposure to antibiotics is viable but not culturable (VBNC) cells. The pulse dosing methodology presented here can, in principle, be applied to such cells, as they can be restored to normal growth and susceptibility to antibiotics upon provision of appropriate stimuli [34,35]. However, experimentally studying this case poses different challenges, as standard cell cultures cannot be routinely used, and this is left for future exploration.”

Figure 2: it is not clear what it is being plotted on the third dimension (i.e. the vertical one that goes from -40 to 20)

- Response: They are the eigenvalues $\lambda_{1},\lambda_{2}$ of the matrix $\mathbf{M}≝\exp\left( \mathbf{A}_{\text{off}}t_{\text{off}} \right)\exp\left( \mathbf{A}_{\text{on}}t_{\text{on}} \right)$
- Action taken: Figure 2 has been redrawn with different view angle, coloring, and vertical axis label, to increase clarity while avoiding cluttering. The figure caption has been adjusted accordingly.

Figure 2. Eigenvalues $\boldsymbol{\lambda}_{\boldsymbol{1}}\boldsymbol{,}\boldsymbol{\lambda}_{\boldsymbol{2}}$ of the matrix $\boldsymbol{M≝}\exp\left( \boldsymbol{A}_{\text{off}}\boldsymbol{t}_{\text{off}} \right)\exp\left( \boldsymbol{A}_{\text{on}}\boldsymbol{t}_{\text{on}} \right)$ governing successive peaks of the bacterial population size, $\boldsymbol{c}\left( \boldsymbol{t}_{\boldsymbol{2}\boldsymbol{n}} \right)$, under pulse dosing with successive on/off pulses of respective durations $\boldsymbol{t}_{\text{on}}\boldsymbol{,}\boldsymbol{t}_{\text{off}}$, given parameter values in Table 1. Values of $\boldsymbol{t}_{\text{on}}\boldsymbol{,}\boldsymbol{t}_{\text{off}}$ that keep both $\boldsymbol{\lambda}_{\boldsymbol{1}}\boldsymbol{,}\boldsymbol{\lambda}_{\boldsymbol{2}}$ below $\boldsymbol{1}$ (equivalently $\ln\left( \boldsymbol{\lambda}_{\boldsymbol{1}} \right)\boldsymbol{,}\ln\left( \boldsymbol{\lambda}_{\boldsymbol{2}} \right)$ below 0) are shown. The white line corresponding to $\left( \boldsymbol{t}_{\text{off}}\boldsymbol{/}\boldsymbol{t}_{\text{on}} \right)_{\boldsymbol{c}}\boldsymbol{\approx2.8}$, eqn. , on the gray plane at 0 indicates the threshold (critical) value of $\boldsymbol{t}_{\text{off}}\boldsymbol{/}\boldsymbol{t}_{\text{on}}$, above which $\boldsymbol{\lambda}_{\boldsymbol{1}}\boldsymbol{>1.}$

Figure 5: the authors should clarify both in the figure and text which antibiotic is being used and what is its concentration.

- Action taken: Both text and figure caption have been changed accordingly as follows.

|   (a) |   (b) |
| --- | --- |

Figure 5. Time-growth (a) and time-kill (Ampicillin at $\boldsymbol{100}\text{μg/mL}$) (b) experiments for generation of data to assess $\boldsymbol{t}_{\text{on}}\boldsymbol{/}\boldsymbol{t}_{\text{off}}$ for design of pulse dosing. 95%-confidence bands are shown. The slope of the cyan straight line (a) indicates the initial slope of the blue line and corresponds to $\boldsymbol{K}_{\boldsymbol{n,}\text{off}}\boldsymbol{/}\ln\left( \boldsymbol{10} \right)$, whereas the blue line slope (b) is $\boldsymbol{K}_{\boldsymbol{n,}\text{on}}\boldsymbol{/}\ln\left( \boldsymbol{10} \right)$.

Why the data in Fig. 5B do not display a biphasic trend typically indicating the presence of persisters? This might have a strong impact on the rest of the study since the authors use this data to extrapolate kon. Could the authors please clarify this important issue.

- Response: Indeed, estimation of $K_{n,\text{on}}$ from the data shown in Figure 5b is an important part of the method presented.
- Action taken: The following text has been added to the revised manuscript: “Note that the pattern shown in Figure 5(b) indicates exponential decline (corresponding to a straight line of the bacterial population logarithm) as the almost horizontal part of the biphasic trend, which is typical of persisters [2], has not yet been reached within 3 hours. That trend is reached through continuation of the experiment after 3 hours, as indicated by the red data points and corresponding bend of the line fitted in Figure 6.”

Lines 219-223: could the authors add statistical analysis to conclude that indeed the two different treatment regimes lead to statistically different outcomes? It looks like there are only 10 CFU/ml in the case of constant dosing

- Response: The non-overlapping 95%-confidence bands for the fitted model outputs shown in the *original* figure already suggest that outcomes are different, but we agree with the Reviewer that further elaboration would be worthwhile.
- Action taken:
  - Figure 6 has been redrawn, to show (a) the separation of 1-standard error (rather than 95% confidence) bands and (b) the projected time for complete reduction of the bacterial population.
  - Figure 6 caption has been modified as “Figure 6. (a) Experimental data and model fit for bacterial population over time, resulting from constant dosing (red) and uniform pulse dosing (blue). The hollow blue circles at 12"h" and "13h" are set to 0 by convention, to indicate undetectable colony forming units (CFU). (b) Fitted model projection for the outcome of constant dosing to reach 0. 68%-confidence bands (1 standard error) are shown.”
  - The following text has been added to Discussion: “The different bactericidal outcomes produced by constant and pulse dosing are evident in Figure 6(a), where the (one-standard-error) confidence bands towards the end of the experimental period of $13\text{h}$ are clearly separated, with pulse dosing yielding no detectable bacterial load and constant dosing leaving a load of about $20\log(\text{CFU/mL})$ at $13\text{h}$. In addition, Figure 6(b) shows the fitted model projection until the bacterial population outcome resulting from constant dosing reaches 0 at about $30\text{h}$, long after pulse dosing has reached the same outcome in less than $12\text{h}$.”

Section 3.4.2 and Figure 8: I found these data confusing since in this case pulse dosing looks far worse than constant dosing. Is this because the pulses are so short? The authors should better clarify this in the manuscript and perhaps compare different pulse dosing regimes next to each other one with advantageous and one with disadvantageous outcome

- Response: Indeed, the Reviewer’s understanding is correct: Pulse dosing is far worse than constant dosing because the ratio $t_{\text{off}}/t_{\text{on}}$ is much larger than suggested by the model.
- Action taken:
  - The corresponding text in section 3.4.2 has been modified to emphasize the point, as follows.
  “In addition to the experimental data in Figure 6, which confirmed rapid bacterial eradication for pulse dosing at ratio $t_{\text{off}}/t_{\text{on}}=2/3$, example cases of pulse dosing at ratios $t_{\text{off}}/t_{\text{on}}$ anticipated by the developed theory and by computer simulations not to result in bacterial eradication were also tested through *in vitro* experiments, as shown in Figure 8. This figure shows the outcome (dots) of two pulse dosing experiments corresponding to the same $t_{\text{off}}/t_{\text{on}}=6$, above the critical value $\left( t_{\text{off}}/t_{\text{on}} \right)_{c}=2.8$ (eqn. (8)), for $t_{\text{on}}=0.5\text{h}$ and $t_{\text{on}}=3\text{h}$. Model predictions (eqns. (1) and (2) with parameter estimates from Table 1 based on data of Figure 6) are also shown.”
  - The caption of Figure 8 has been modified to emphasize the point, as follows.
  “Figure 8. Experimental values (dots) and model predictions (lines) for constant dosing (red) and pulse dosing with $t_{\text{off}}/t_{\text{on}}=6>\left( t_{\text{off}}/t_{\text{on}} \right)_{c}$ (blue) for (a) $\left\{ t_{\text{on}}, t_{\text{off}} \right\}=\left\{ 0.5\text{h}, 3\text{h} \right\}$ and (b) $\left\{ t_{\text{on}}, t_{\text{off}} \right\}=\left\{ 3\text{h}, 18\text{h} \right\}$.”
  - The text in Introduction has been modified to emphasize the point of experimentally confirming both positive and negative predictions for pulse dosing (bacterial eradication or not, respectively), as follows.
  “and (c) experimental confirmation in vitro of both positive and negative model predictions (bacterial eradication or not, respectively).”

Discussion
In its present state the discussion reads more as a summary than putting this new manuscript in the context of the persister research field. The authors could compare their findings with existing data in the literature, including the studies suggested above.

- Action taken:
  - Subsection “4.1 Summary of results” was added to the Discussion section, to provide a summary of the flow of reasoning and results obtained in the manuscript.
  - Subsection “4.4 Application of the proposed theory to literature data” and Tables 2 and 3 were added to the Discussion section, to provide a quantitative comparison between predictions of the method developed in the manuscript and literature findings.

# Reviewer #3:

The authors used a simple mathematical model to study the optimal pulsing strategy to apply antibiotics and eliminate persister bacterial cells.
I believe this is a valid and interesting contribution to the field.
My main concerns are:
1- the type of plots used in figures 2, 3, 4 and 11 are difficult to interpret. I would check if series of 2-dimensional plots could be more easily interpretable than the current plots.

- Response: As each of the variables plotted depends on two independent variables, we would prefer to retain the 3D structure of these plots, to convey the intended message. We considered contour plots (2D with color showing the third D) but found them less intuitive. We also considered plotting for each dependent variable a family of curves that would show dependence on the first independent variable on the horizontal axis and on the second independent variable by the curve number in the family. We found this less intuitive as well, and opted instead for addition of a rectangular grid in white to each surface (in addition to the constant-latitude lines mesh in black).
- Action taken: All four plots and corresponding captions have been revised for clarity.

2- The authors used in vitro experimental data to fit parameter values. If a is 0 both in in the antibiotic on and off states, how are the persister cells generated?

- Response: There is a small initial fraction of persister cells, which dominate as the source in the pool for all persisters.
- Action taken: A comment to that effect was added as follows: “…, in agreement with the assumption $a\approx0,$ i.e. that persisters can be sourced to the initial pool of cells.”

3- As the model parameters can change with bacterial species and with the type of antibiotic, it would be useful to present a sensitivity analysis showing how the peak to peak decay rate varies with the different parameters.

- Response: Indeed, this is shown in Figure 3. An important point of that figure is that the optimal ratio $\left( t_{\text{on}}/t_{\text{off}} \right)_{\text{opt}}$ depends on $R_{p}≝\frac{K_{p,\text{on}}}{K_{p,\text{off}}}, R_{n}≝\frac{K_{n,\text{on}}}{K_{n,\text{off}}}$, rather than on the individual values of $K_{p,\text{on}}, K_{p,\text{off}},K_{n,\text{on}},K_{n,\text{off}}.$
- Action taken: A corresponding comment was added to the text of the revised manuscript, as follows.
  “Note that $\left( t_{\text{off}}/t_{\text{on}} \right)_{\text{opt}}$ in eqn. (11) depends on the ratios $R_{p}≝K_{p,\text{on}}/K_{p,\text{off}}, R_{n}≝K_{n,\text{on}}/K_{n,\text{off}}$, rather than on the individual values of $K_{p,\text{on}}, K_{p,\text{off}},K_{n,\text{on}},K_{n,\text{off}}.$”

4- The model describes well the decay of bacterial populations in vitro. The authors should discuss what is different with the population dynamics in vivo (during an infection), and if the model needs to be adapted in that scenario.

- Response: An excellent point. We address this issue in section 5 (Conclusions and Future Work)
- Action taken: Added bullet point “Ultimately in vivo studies”
